# Early red nucleus atrophy in relapse‐onset multiple sclerosis

**DOI:** 10.1002/hbm.25213

**Published:** 2020-10-13

**Authors:** Monica Margoni, Davide Poggiali, Sofia Zywicki, Martina Rubin, Andrea Lazzarotto, Silvia Franciotta, Maria Giulia Anglani, Francesco Causin, Francesca Rinaldi, Paola Perini, Massimo Filippi, Paolo Gallo

**Affiliations:** ^1^ Multiple Sclerosis Centre of the Veneto Region (CeSMuV) University Hospital of Padua Padua Italy; ^2^ Padova Neuroscience Centre (PNC) University of Padua Padua Italy; ^3^ Department of Mathematics University of Padua Padua Italy; ^4^ Neuroradiology Unit University Hospital of Padua Padua Italy; ^5^ Neuroimaging Research Unit, Institute of Experimental Neurology, Division of Neuroscience IRCCS San Raffaele Scientific Institute Milan Italy; ^6^ Neurology Unit IRCCS San Raffaele Scientific Institute Milan Italy; ^7^ Neurophysiology Unit IRCCS San Raffaele Scientific Institute Milan Italy; ^8^ Vita‐Salute San Raffaele University Milan Italy; ^9^ Department of Neurosciences, Medical School University of Padua Padua Italy

**Keywords:** cerebellum, MRI, red nucleus

## Abstract

No study has investigated red nucleus (RN) atrophy in multiple sclerosis (MS) despite cerebellum and its connections are elective sites of MS‐related pathology. In this study, we explore RN atrophy in early MS phases and its association with cerebellar damage (focal lesions and atrophy) and physical disability. Thirty‐seven relapse‐onset MS (RMS) patients having mean age of 35.6 ± 8.5 (18–56) years and mean disease duration of 1.1 ± 1.5 (0–5) years, and 36 age‐ and sex‐matched healthy controls (HC) were studied. Cerebellar and RN lesions and volumes were analyzed on 3 T‐MRI images. RMS did not differ from HC in cerebellar lobe volumes but significantly differed in both right (107.84 ± 13.95 mm^3^ vs. 99.37 ± 11.53 mm^3^, *p* = .019) and left (109.71 ± 14.94 mm^3^ vs. 100.47 ± 15.78 mm^3^, *p* = .020) RN volumes. Cerebellar white matter lesion volume (WMLV) inversely correlated with both right and left RN volumes (*r* = −.333, *p* = .004 and *r* = −.298, *p* = .010, respectively), while no correlation was detected between RN volumes and mean cortical thickness, cerebellar gray matter lesion volume, and supratentorial WMLV (right RN: *r* = −.147, *p* = .216; left RN: *r* = −.153, *p* = .196). Right, but not left, RN volume inversely correlated with midbrain WMLV (*r* = −.310, *p* = .008), while no correlation was observed between whole brainstem WMLV and either RN volumes (right RN: *r* = −.164, *p* = .164; left RN: *r* = −.64, *p* = .588). Finally, left RN volume correlated with vermis VIIb (*r* = .297, *p* = .011) and right interposed nucleus (*r* = .249, *p* = .034) volumes. We observed RN atrophy in early RMS, likely resulting from anterograde axonal degeneration starting in cerebellar and midbrain WML. RN atrophy seems a promising marker of neurodegeneration and/or cerebellar damage in RMS.

## Graphical abstract

The cerebellum and the brainstem are major sites of pathology in multiple sclerosis (MS) and their involvement, although frequently asymptomatic, is known to be associated with a more severe prognosis. We explored RN atrophy in early MS phases and its association with cerebellar damage and physical disability. We observed RN atrophy in early relapse‐onset (RMS), likely resulting from anterograde axonal degeneration starting in cerebellar and midbrain WML. RN atrophy seems a promising marker of neurodegeneration and/or cerebellar damage in RMS.

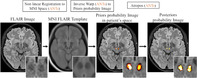

## 1 INTRODUCTION

The red nucleus (RN), a large neuronal structure located in the most rostral part of ventral midbrain, owes its name to its high iron content, which makes it clearly identifiable both in fresh tissue sections and in T2‐weighted magnetic resonance imaging (MRI) sequences. According to its cytoarchitecture, the RN is divided in a rostral parvocellular part (pRN), accounting for up to 80% of the nucleus volume and receiving inputs primarily from the nucleus dentatus, and a caudal magnocellular part (mRN, 20%) receiving inputs primarily from nucleus interpositus and nucleus emboliform (Massion, 1988; Onodera & Hicks, 2009; ten Donkelaar, 1988).

mRN and pRN have different functional roles. While mRN is involved in intra‐ and inter‐limb coordination (Lavoie & Drew, 2002) and probably plays a role in compensating lesions of the corticospinal tract (Belhaj‐Saif & Cheney, 2000; Siegel, Fink, Strittmatter, & Cafferty, 2015), pRN has been suggested to be involved in more complex functional networks, including those integrating cognitive‐motor functions such as motor learning, error encoding, timing, and control of the ongoing movement (Habas, Guillevin, & Abanou, 2010; Lang et al., 2017; Reid et al., 2009). These observations further point out the association of specific cerebellar lobules with cognitive and affective functions (Koziol et al., 2014; Lazzarotto et al., 2020; Schmahmann & Sherman, 1997). Finally, worth of interest is also the possible role of cerebellar and RN damage in the early development of disabling multiple sclerosis (MS) symptoms such as fatigue and depression (Lazzarotto et al., 2020).

The cerebellum and the brainstem are major sites of pathology in MS and their involvement, although frequently asymptomatic, is known to be associated with a more severe prognosis (Damasceno, Von Glehn, Brandao, Damasceno, & Cendes, 2013; Tintore et al., 2010). Lesions damaging RN structure and connections may cause severe clinical picture, such as the rubral tremor (Koch, Mostert, Heersema, & De Keyser, 2007). Due to its major connections with the cerebellum, RN atrophy can result from trans‐synaptic degeneration following cerebellar damage. Indeed, in a preclinical study in mutant mice, the loss of Purkinje cells was followed by the progressive atrophy of deep cerebellar nuclei due to anterograde trans‐synaptic degeneration (Triarhou, Norton, & Ghetti, 1987).

Given these premises, we aimed to explore whether RN atrophy can be detected in the early disease phases, and whether this is associated with cerebral or cerebellar MRI metrics of white matter (WM) or gray matter (GM) damage, and early physical disability in relapse‐onset MS (RMS) patients.

## 2 MATERIALS AND METHODS

### 2.1 Patients

Thirty‐seven relapsing–remitting (RRMS) patients (mean age: 35.6 ± 8.5 years; range: 18–56 years; F/M: 25/12) who met the 2010 and 2017 diagnostic criteria (Polman et al., 2011; Thompson et al., 2018) were enrolled in this retrospective study. Thirty‐six sex‐ and age‐matched healthy controls (HC) (mean age: 33.8 ± 9.9 years; range: 23–63 years; F/M = 24/12) constituted the reference population.

All patients that were diagnosed to have MS in the period March 2014 to August 2018, were selected according to the following criteria: (a) age range, 18–65 years; (b) clinical disease duration <5 years; (c) no history/evidence of neurologic or psychiatric disorders other than MS; (d) no history of alcohol or drug abuse; (e) good quality of MRI (i.e., we excluded those with movement artifacts).

Each participant gave written informed consent and the study was approved by the local Ethics Committee, according to the IV revision of Declaration of Helsinki.

### 2.2 Clinical assessment

All patients underwent a neurological examination, including Expanded Disability Status Scale (EDSS) (Kurtzke, 1983) within 1 week from the MRI acquisition.

### 2.3 MRI data acquisition

MRI was obtained on a 3.0 T scanner (Ingenia, Philips Medical Systems, Best, The Netherlands) with 33 mT/m power gradient and a 32‐channel head coil. No major hardware upgrades occurred during the study, and bimonthly quality assurance sessions assured measurement stability. The MRI protocol included the following sequences: (a) three‐dimensional (3D) T1 TFE: repetition time (RT) = 7,2, echo time (ET) = 3,3, TFE factor 218; 165 contiguous axial slices with the off‐center positioned on zero with thickness of 1.0 mm; flip angle = 9; matrix size = 220 × 218; FOV 240 × 240 × 181.5 mm; (b) 3D‐FLAIR: RT = 4,800 ms, ET = 293 ms, inversion time (IT) = 1,650 ms; 331 contiguous axial slices with thickness of 1.0 mm; matrix size 220 × 217; and FOV = 240 × 240 × 182 mm; (c) 3D‐DIR: RT = 5,500 ms, ET = 294 ms, inversion time (IT) = 2,550 ms; 281 contiguous axial slices with thickness of 1.0 mm; matrix size 208 × 209; and FOV = 250 × 250 × 168 mm.

### 2.4 MRI data processing

Quantification of both WM lesion volume (WMLV) on FLAIR images and GM lesion volume (GMLV) on DIR images was performed in each patient by two experienced observers (SZ, MR), unaware of subject identity, employing a semiautomated segmentation technique (ITKSNAP).

Mean cortical thickness and intracranial volume (ICV) were obtained by applying the Freesurfer software on lesion‐filled 3D‐T1 weighted images (Fischl & Dale, 2000).

Cerebellar volumes were calculated on lesion‐filled 3DT1‐weighted images using the spatially unbiased infratentorial toolbox (SUIT) version 3.2, implemented in statistical parametric mapping 12 (SPM 12) (software: http://www.fil.ion. ucl.ac.uk/spm).

Cerebellum and brainstem were identified and isolated automatically, and a mask was automatically generated by calculating the maximum of the probability of each voxel belonging to one of these structures. Each obtained mask was visually inspected. Next, the isolated cerebellum was aligned to the SUIT atlas template via an affine transformation (for the linear part of the normalization) and a nonlinear transformation using the diffeomorphic anatomical registration using Exponentiated Lie algebra (Avants et al., 2011). The individual cerebellum was therefore resliced in the atlas space, modulating in order to grant volume preservation (D'Ambrosio et al., 2017). Finally, by applying an inverse transformation matrix derived from the previous co‐registration step, the SUIT atlas was aligned to the native subject space, and lobular volumes were calculated.

RN volumes were obtained from FLAIR images using a FLAIR template in MNI152 space, derived from a sex and age‐matched healthy population. Priors probability images of RN, manually labeled on high resolution MRIs of a healthy population (*n* = 44, F/M = 21/23, mean age: 32.9, range: 18–60), were also employed (Winkler, Kochunov, & Glahn, n.d.; Ashburner & Friston, 2005; Keuken et al., 2014; Keuken et al., 2017; Kochunov et al., 2006).

**FIGURE 1:**
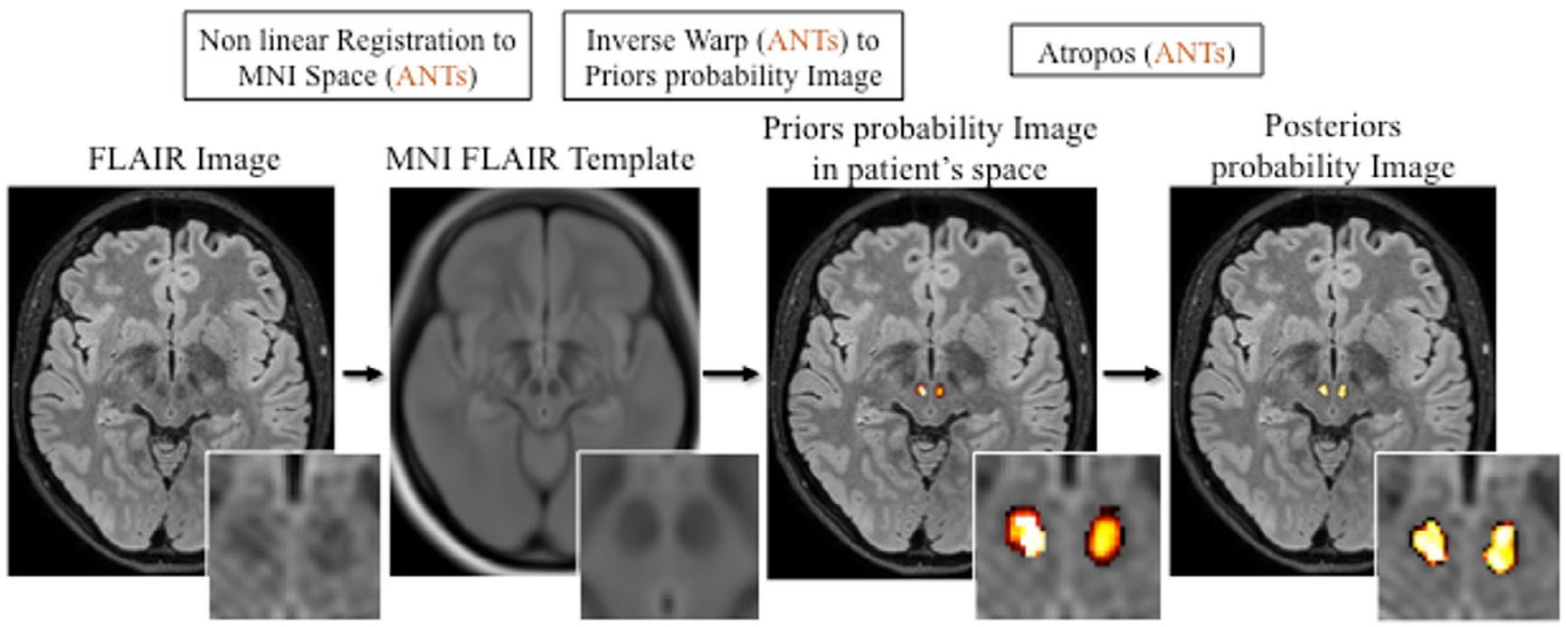
A schematic illustration of the processing pipeline to evaluate red nucleus volume (see Material and Methods for explanations)

FLAIR images were nonlinearly registered to the FLAIR template by means of ANTs registration tool (Avants, Epstein, Grossman, & Gee, 2008), and the inverse warp was applied to priors. From the so‐obtained priors in patient's space, the posteriors probability images were computed by means of Atropos (Wang & Yushkevich, 2013) (Figure 1).

Two experienced investigators (MM, DP), blinded to clinical data, visually inspected all RN. Only in three cases the pipeline was re‐run due to inconsistent segmentation.

## 3 STATISTICAL ANALYSIS

Statistical analysis was performed using SPSS Statistics 23 (SPSS Chicago, Ilinois). A Shaphiro‐Wilk test and visual analysis of histograms were used to test the normality of the data.

Student's *t* test was used to test group differences in term of age, χ^2^ test was used to test possible differences in terms of sex.

Group differences in terms of cerebellar and RN volumes were tested via multivariate Generalized Linear Model (GLM), including ICV as a covariate of no interest to account for head size. Correlations between MRI metrics and clinical disability were explored with Spearman's rank correlation coefficient.

All p values were two‐sided and considered statistically significant when *p* < .05. Since our study is exploratory, we did not adjust for multiple comparisons.

## 4 RESULTS

### 4.1 Clinical and MRI findings in RRMS and HC

RRMS and HC did not differ in age (*p* = .42) and sex (*p* = 1.0). At MRI scan, the patients had mean disease duration of 1.1 ± 1.5 years (range 0–5) and median EDSS score of 2.0 (interquartile range [IQR]: 1.5–2.0, range: 1.0–6.0). The median cerebellar, brainstem and pyramidal functional system (FS) scores were 0.0 (IQR 0.0–0.0; range: 0.0–2.0), 0.0 (IQR 0.0–0.0: range: 0.0–2.0) and 1.0 (IQR 1.0–1.0; range: 0.0–3.0), respectively.

RRMS had a mean supratentorial white matter lesion volume (WMLV) of 341.02 ± 1,172.80 mm^3^, cerebellar WMLV of 123.13 ± 163.43 mm^3^, brainstem WMLV of 85.08 ± 147.0 mm^3^, midbrain WMLV of 25.13 ± 56.45 mm^3^ and cortical gray matter lesion volume (GMLV) of 59.65 ± 214.43 mm^3^. Mean cortical thickness did not differ between RRMS and HC (5.78 ± 0.27 mm vs. 5.87 ± 0.38 mm, *p* = .371).

Table 1 summarizes the main demographic, clinical and MRI characteristics of the groups.

**TABLE 1:** Demographic and clinical features of the groups included in the study

	HC (*n* = 36)	RRMS (*n* = 37)	*p* *
Sex, F/M	24/12	25/12	1.0
Age at MRI scan, mean ± *SD* (years)	33.8 ± 9.9	35.6 ± 8.5	.42
Disease duration, mean ± *SD* (years)	–	1.1 ± 1.5	–
EDSS, median (IQR)	–	2.0 (1.5–2.0)	–
Right RN, mean ± *SD* (mm^3^)	107.84 ± 13.95	99.37 ± 11.53	*.019* *
Left RN, mean ± *SD* (mm^3^)	109.71 ± 14.94	100.47 ± 15.78	*.020* *
Mean cortical thickness (mm)	5.87 ± 0.38	5.78 ± 0.27	.371
Cerebellar WMLV, mean ± *SD* (mm^3^)	–	123.13 ± 163.43	–
Supratentorial WMLV, mean ± *SD* (mm^3^)	–	341.02 ± 1,172.80	–
Brainstem WMLV, mean ± *SD* (mm^3^)	–	85.08 ± 147.0	–
Midbrain WMVL, mean ± *SD* (mm^3^)	–	25.13 ± 56.45	–
Cortical GMLV, mean ± *SD* (mm^3^)		59.65 ± 214.43	–

Abbreviations: EDSS, expanded disability status scale; GMLV, gray matter lesion volume; HC, healthy controls; IQR, interquartile range; RN, red nucleus; RRMS, relapsing–remitting multiple sclerosis; *SD*, standard deviation; WMLV, white matter lesion volume.

* *p* < .05.

### 4.2 RN volume is decreased in RRMS compared with HC

Anterior and posterior cerebellar lobe volumes did not differ between MS patients and HC. Despite the very short disease duration, MS patients had a significant reduction of both right (*p* = .019; 107.84 ± 13.95 mm^3^ vs. 99.37 ± 11.53 mm^3^) and left RN (*p* = .020; 109.71 ± 14.94 mm^3^ vs. 100.47 ± 15.78 mm^3^) volume compared with HC (Table 1). A complete list of the cerebellar volumetric analysis, with the respective *p* values, is reported in Table 2.

**TABLE 2:** Mean cerebellar volumes (mm^3^) and *SD* of RRMS and healthy controls (HC)

	HC (*n* = 36)		RRMS (*n* = 37)		
	Mean	*SD*	Mean	*SD*	*p*
**Left I‐IV**	0.002301502	0.000670027	0.002376645	0.000561846	.614
**Right I‐IV**	0.002590352	0.000842328	0.002791260	0.000681491	.311
**Left V**	0.002919112	0.000872980	0.003009530	0.000723322	.628
**Right V**	0.002740069	0.000927967	0.002935002	0.000692913	.298
**Left VI**	0.006317410	0.002007192	0.006480943	0.001637477	.724
**Vermis VI**	0.001424024	0.000449293	0.001430352	0.000349973	.973
**Right VI**	0.005369853	0.001606742	0.005707507	0.001348242	.374
**Left crus I**	0.009709009	0.002741510	0.009378255	0.002320982	.512
**Vermis crus I**	1.06528E‐05	5.61335E‐06	1.10246E‐05	6.81924E‐06	1.000
**Right crus I**	0.008383129	0.002303089	0.008636830	0.001948583	.705
**Left crus II**	0.006719993	0.002055345	0.006909523	0.001895466	.769
**Vermis crus II**	0.000351424	0.000101609	0.000338894	8.20934E‐05	.595
**Right crus II**	0.006164449	0.001827956	0.006507936	0.001707451	.487
**Left VIIb**	0.003326942	0.001027895	0.003502277	0.000866804	.455
**Vermis VIIb**	0.000160291	5.13925E‐05	0.000150075	4.06692E‐05	.480
**Right VIIb**	0.003379965	0.001037441	0.003513652	0.000856862	.564
**Left VIIIa**	0.003382789	0.001072485	0.003625168	0.000891045	.327
**Vermis VIIIa**	0.000891382	0.000258704	0.000861586	0.000186131	.558
**Right VIIIa**	0.003322524	0.001090567	0.003514819	0.000879843	.440
**Left VIIIb**	0.002637954	0.000846047	0.002854016	0.000777884	.274
**Vermis VIIIb**	0.000443174	0.000128391	0.000455487	9.50461E‐05	.685
**Right VIIIb**	0.002931313	0.000941996	0.003119420	0.000791151	.390
**Left IX**	0.002375767	0.000705235	0.002399400	0.00059463	.771
**Vermis IX**	0.000560461	0.000139537	0.000567502	0.000113259	.773
**Right IX**	0.002854704	0.000838599	0.002868548	0.000646645	.878
**Left X**	0.000363149	0.000127645	0.000389401	0.000138833	.305
**Vermis X**	0.000275069	6.7564E‐05	0.000292240	6.6236E‐05	.211
**Right X**	0.000431827	0.000139280	0.000466224	0.000120889	.242
**Left dentate**	0.000568435	0.000125495	0.00057703	0.000115679	.660
**Right dentate**	0.000546634	0.000147238	0.000538615	0.000106714	.910
**Left interposed**	9.126E‐06	3.7176E‐06	8.17138E‐06	3.23017E‐06	.200
**Right interposed**	5.60918E‐06	2.51593E‐06	5.56481E‐06	2.32856E‐06	1.000

*Note: p* values were calculated with Generalized Linear Model; significance level was set at *p* < .05.

### 4.3 RN volume correlates with cerebellar and midbrain WM lesions

A significant inverse correlation was found between cerebellar WMLV and both RN volumes (right: *r* = −.333, *p* = .004, and left: *r* = −.298, *p* = .010, respectively), while no correlation could be detected between RN volumes and mean cortical thickness (left RN: *r* = .155, *p* = .190; right RN: *r* = .177, *p* = .134), cerebellar GMLV (left RN: *r* = −.125, *p* = .291, right RN: *r* = −.227, *p* = .053) and supratentorial WMLV (right RN: *r* = −.147, *p* = .216; left RN: *r* = −.153, *p* = .196, respectively). Right, but not left, RN volume inversely correlated with midbrain WMLV (*r* = −.310, *p* = .008), while no correlation was observed between brainstem WMLV and either RN volumes (right RN: *r* = −.164, *p* = .164; left RN: *r* = −.64, *p* = .588).

### 4.4 RN atrophy associates with cerebellar lobule atrophy and cerebellar function

Some low/moderate, but significant, correlations emerged between the volume of RN volume and that of some cerebellar lobules. Namely, the left RN volume correlated with vermis VIIb (*r* = .297, *p* = .011) and right interposed nucleus (*r* = .249, *p* = .034) volumes. Moreover, the right RN volume correlated with cerebellar FS score of EDSS (*r* = −.360, *p* = .029).

No correlation was found between RN volumes and disease duration (right RN: *r* = −.318, *p* = .055; left RN: *r* = −.274, *p* = .101) or EDSS score (right RN: *r* = −.168, *p* = .444; left RN: *r* = −.09, *p* = .679).

## 5 DISCUSSION

Looking for early markers of neurodegeneration in RMS, we explored RN changes in a very early disease phase. Since the cerebellum and its connections are major sites of MS‐related pathology (Parmar et al., 2018) and a pre‐clinical study in Purkinje cell degeneration mutant mice disclosed trans‐synaptic degeneration of deep cerebellar nuclei (Triarhou et al., 1987), our working hypothesis was that early RN atrophy could be the result of trans‐synaptic anterograde axonal neurodegeneration starting in cerebellar WM and GM lesions.

We observed that RN volume in RMS, having very short disease duration (1.1 ± 1.5 years), was significantly lower compared with matched HC and correlated with cerebellar and midbrain WMLV, but not with supratentorial WMLV. Moreover, RN volume directly associated with the volume of some cerebellar lobules, but not with cerebellar GMLV. These observations suggest that RN atrophy could, at least partly, result from an axonal degeneration starting in cerebellum and midbrain WM lesions.

Our findings are particularly interesting on the light of previous observations in other neurological diseases. Indeed, RN volume was increased in patients with Parkinson's disease and significantly correlated with disease duration and severity, suggesting a compensatory activation of this nucleus (Colpan & Slavin, 2010), probably aimed at compensating the impaired function of the extra‐pyramidal network. Moreover, in nonhuman primates (Philippens, Wubben, Franke, Hofman, & Langermans, 2019) larger RN volume inversely associated with the severity of extra‐pyramidal symptoms. Finally, a damaged striato‐thalamo‐cortical pathway was found to result in a compensatory increase in RN activation (Philippens et al., 2019). All these observations suggest a compensatory function of RN in the presence of an extra‐pyramidal disorder.

The RN seems to play a compensatory role even in patients with severe corticospinal tract injury following ischaemic stroke. Indeed, elevated levels of neuronal activity were observed in the RN of the affected hemisphere, especially in the early phases following stroke, suggesting that RN could work as a compensatory structure to support the residual motor functions (Yeo & Jang, 2010). Microstructural RN changes, reflecting plastic and functional remodeling and associated with better motor function recovery, have been confirmed in other studies (Takenobu et al., 2014).

The above‐summarized findings in Parkinson's disease and stroke strongly differ from our observations. Indeed, while direct pyramidal and extra‐pyramidal damages are followed by a compensatory reaction of the RN, cerebellar damage associates with its atrophy. Thus, the initial RN atrophy observed in early RMS phases, may be considered a potential MRI marker of neurodegeneration following cerebellar damage and merits to be further investigated as negative prognostic factor. Indeed, the early NR atrophy might impair its potential functional compensatory role in case of corticospinal damage, that is, one of the major determinants of disability in MS.

Our data are in line with the increasing evidence of atrophy of deep gray matter nuclei in early RMS stages (Eshaghi et al., 2018), which indicates that this MRI metric may have a prognostic value since it associates with an increased risk of EDSS progression (Eshaghi et al., 2018) and cognitive decline in a relative shorter time (Riccitelli et al., 2011; Schoonheim et al., 2015). Interestingly, we also found an inverse association between RN atrophy and the cerebellar FS score of EDSS, suggesting that NR atrophy may be a specific marker of cerebellar damage rather than a generic marker of diffuse neurodegeneration.

**FIGURE 2:**
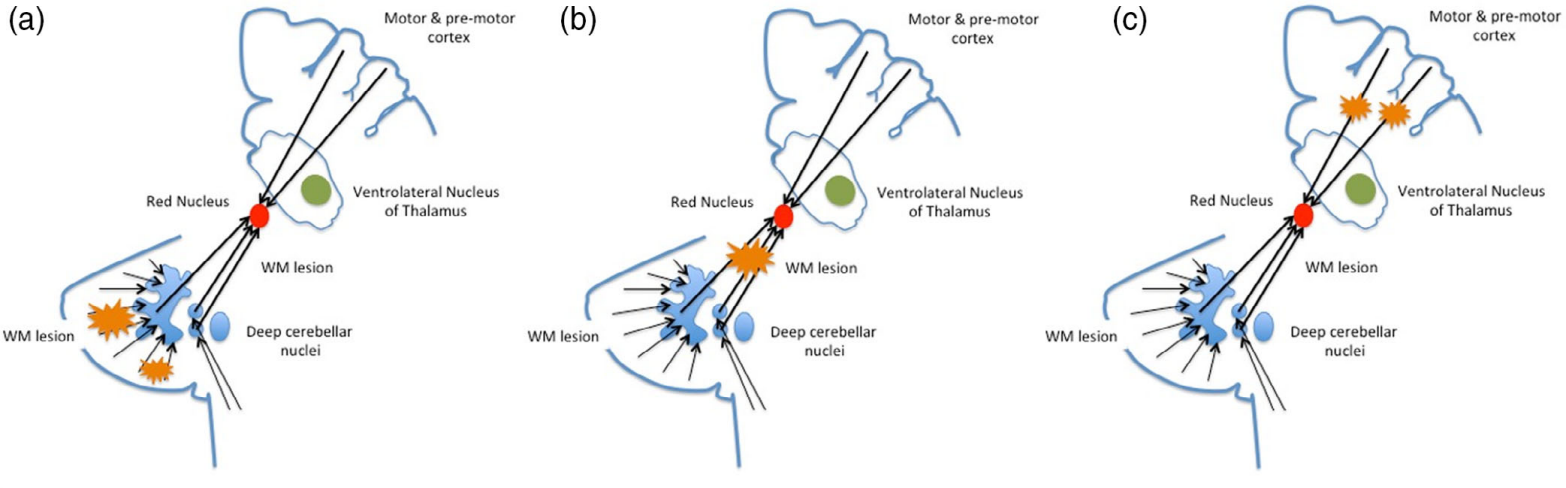
Possible causes of anterograde trans‐synaptic degeneration of red nucleus (RN) RMS: (a) lesions in the cerebellar subcortical white matter (WM); (b) WM damage between the cerebellar nuclei and RN (i.e., superior cerebellar peduncle); (c) WM damage of the cortical‐rubral trait

The initial and significant, although slight correlations between RN atrophy and some cerebellar lobule atrophy indicates that the loss of volumes of these structures may proceed in parallel, probably as a consequence of retrograde and trans‐synaptic anterograde axonal degenerations starting in cerebellar WM lesions (Figure 2). Consequently, we may argue that RN atrophy could also reflect cerebellar pathology in normal appearing cerebellar white and gray matter, not detectable by conventional MRI sequences.

We are aware of the major limitation of our explorative study, namely, the relative low number of patients analyzed. However, we would like to further point out the single center and blind setting of our study, that make our data homogeneous and reliable. Moreover, we are also aware that only longitudinal studies may help to draw conclusions on the clinical hypothetic prognostic value of RN. Thus, we intend to follow our cohort of patients in order to verify this hypothesis.

Concluding, we found evidence of RN atrophy in very early stages of RMS and its association with cerebellar and midbrain WMLV and selective cerebellar lobule atrophy. RN atrophy is worthy of consideration as a promising marker of subclinical cerebellar damage and neurodegeneration in RMS. The prognostic value of RN atrophy merits to be explored given the relative simplicity with which this nucleus can be visualized and measured on routinely available MRI sequences.

### DISCLOSURE OF INTERESTS

Monica Margoni reports grants from Genzyme Sanofi, Merck Serono, Biogen Idec, grants and personal fees from Novartis, during the conduct of the study. Sofia Zywicki, Davide Poggiali, Martina Rubin have nothing to disclose. Alice Riccardi reports personal fees from Mylan, Biogen Idec, Genzyme Sanofi. Francesca Rinaldi reports grants and personal fees from Genzyme Sanofi, Merck Serono, Biogen Idec, grants from Novartis, during the conduct of the study. Paola Perini reports grants and personal fees from Merck Serono, Biogen Idec, Genzyme Sanofi, Roche, Novartis, during the conduct of the study. Massimo Filippi is Editor‐in‐Chief of the Journal of Neurology; received compensation for consulting services and/or speaking activities from Bayer, Biogen Idec, Merck‐Serono, Novartis, Roche, Sanofi Genzyme, Takeda, and Teva Pharmaceutical Industries; and receives research support from Biogen Idec, Merck‐Serono, Novartis, Roche, Teva Pharmaceutical Industries, Italian Ministry of Health, Fondazione Italiana Sclerosi Multipla, and ARiSLA (Fondazione Italiana di Ricerca per la SLA). Paolo Gallo reports grants and personal fees from Merck Serono, Biogen Idec, Genzyme Sanofi, grants and personal fees from Novartis, grants from University of Padua, Department of Neurosciences DNS, grants from Veneto Region of Italy, grants from Italian Association for Multiple Sclerosis (AISM), grants from Italian Ministry of Public Health, during the conduct of the study.

## Data Availability

The data that support the findings of this study are available from the corresponding author upon reasonable request.
